# False lumen pressure estimation in type B aortic dissection using 4D flow cardiovascular magnetic resonance: comparisons with aortic growth

**DOI:** 10.1186/s12968-021-00741-4

**Published:** 2021-05-13

**Authors:** David Marlevi, Julio A. Sotelo, Ross Grogan-Kaylor, Yunus Ahmed, Sergio Uribe, Himanshu J. Patel, Elazer R. Edelman, David A. Nordsletten, Nicholas S. Burris

**Affiliations:** 1 Institute for Medical Engineering and Science, Massachusetts Institute of Technology, Cambridge, MA USA; 2 School of Biomedical Engineering, Universidad de Valparaíso, Valparaíso, Chile; 3 Biomedical Imaging Center, Pontificia Universidad Católica de Chile, Santiago, Chile; 4 ANID-Millennium Science Initiative Program-Millennium Nucleus in Cardiovascular Magnetic Resonance, Santiago, Chile; 5 Department of Radiology, Schools of Medicine, Pontificia Universidad Católica de Chile, Santiago, Chile; 6 Department of Biomedical Engineering, University of Michigan, Ann Arbor, MI USA; 7 Department of Cardiac Surgery, University of Michigan, Ann Arbor, MI USA; 8 School of Biomedical Engineering and Imaging Sciences, King’s College London, London, UK; 9 Department of Radiology, University of Michigan, 1500 E. Medical Center Drive, Cardiovascular Center 5588, SPC-5030, Ann Arbor, MI 48109-5030 USA

**Keywords:** Type B aortic dissection, 4D flow magnetic resonance imaging, 4D flow MRI, False lumen, Aortic growth rate, False lumen ejection fraction, Maximum systolic deceleration rate, Relative pressure

## Abstract

**Background:**

Chronic type B aortic dissection (TBAD) is associated with poor long-term outcome, and accurate risk stratification tools remain lacking. Pressurization of the false lumen (FL) has been recognized as central in promoting aortic growth. Several surrogate imaging-based metrics have been proposed to assess FL hemodynamics; however, their relationship to enlarging aortic dimensions remains unclear. We investigated the association between aortic growth and three cardiovascular magnetic resonance (CMR)-derived metrics of FL pressurization: false lumen ejection fraction (FLEF), maximum systolic deceleration rate (MSDR), and FL relative pressure (FL ΔP_max_).

**Methods:**

**C**MR/CMR angiography was performed in 12 patients with chronic dissection of the descending thoracoabdominal aorta, including contrast-enhanced CMR angiography and time-resolved three-dimensional phase-contrast CMR (4D Flow CMR). Aortic growth rate was calculated as the change in maximal aortic diameter between baseline and follow-up imaging studies over the time interval, with patients categorized as having either ‘stable’ (< 3 mm/year) or ‘enlarging’ (≥ 3 mm/year) growth. Three metrics relating to FL pressurization were defined as: (1) FLEF: the ratio between retrograde and antegrade flow at the TBAD entry tear, (2) MSDR: the absolute difference between maximum and minimum systolic acceleration in the proximal FL, and (3) FL ΔP_max_: the difference in absolute pressure between aortic root and distal FL.

**Results:**

FLEF was higher in enlarging TBAD (49.0 ± 17.9% vs. 10.0 ± 11.9%, p = 0.002), whereas FL ΔP_max_ was lower (32.2 ± 10.8 vs. 57.2 ± 12.5 mmHg/m, p = 0.017). MSDR and conventional anatomic variables did not differ significantly between groups. FLEF showed positive (r = 0.78, p = 0.003) correlation with aortic growth rate whereas FL ΔP_max_ showed negative correlation (r = − 0.64, p = 0.026). FLEF and FL ΔP_max_ remained as independent predictors of aortic growth rate after adjusting for baseline aortic diameter.

**Conclusion:**

Comparative analysis of three 4D flow CMR metrics of TBAD FL pressurization demonstrated that those that focusing on retrograde flow (FLEF) and relative pressure (FL ΔP_max_) independently correlated with growth and differentiated patients with enlarging and stable descending aortic dissections. These results emphasize the highly variable nature of aortic hemodynamics in TBAD patients, and suggest that 4D Flow CMR derived metrics of FL pressurization may be useful to separate patients at highest and lowest risk for progressive aortic growth and complications.

**Supplementary Information:**

The online version contains supplementary material available at 10.1186/s12968-021-00741-4.

## Introduction

Chronic type B aortic dissection (TBAD) is characterized by high incidence of long-term complications and elevated mortality [1], with progressive growth of the false lumen (FL) being a primary contributor to adverse outcomes [2]. Thoracic endovascular aortic repair (TEVAR) has been increasingly used to reduce aneurysm formation in TBAD [3, 4], however, TEVAR does not yield favorable results in all patients, particularly those with chronic dissection, and comes with procedural risks [5, 6]. Thus, there is a significant need for better techniques to assess risk of FL growth to identify patients who may benefit most from prophylactic repair.

Current methods for predicting growth in TBAD are based on anatomic features, most commonly maximal aortic diameter [7]. However, such evaluation does not include assessment of abnormal blood flow [7–9], which is believed to play an important role in driving aortic growth. An increasing body of experimental evidence [10–17] has demonstrated that an excess of FL inflow relative to outflow leads to increasing pressurization of the FL, which promotes growth due to elevated stresses on the weakened aortic wall. Despite the importance of FL pressurization, current techniques to measure FL pressures require invasive catheterization, which is potentially hazardous and rarely performed clinically. Thus, there remains a significant need for clinically applicable techniques to quantify FL pressure and hemodynamic abnormalities in vivo to advance translation of these experimental findings to clinical care of TBAD patients.

Time-resolved three-dimensional phase-contrast cardiovacular magnetic resonance (4D Flow CMR) is a non-invasive technique that provides volumetric assessment of blood flow, and has been extensively applied to study aortic hemodynamics [18–23]. Recent 4D Flow CMR studies have also proposed ways of estimating FL pressurization and to predict growth in TBAD [18–20, 24]. Burris et al. [18] proposed FL ejection fraction (FLEF)—the ratio between retrograde and antegrade flow through the FL opening—as indicative of pressurization, linking FLEF to TBAD growth. Similarly, Ruiz Munoz et al. [24] used 4D flow CMR to measure the maximum systolic deceleration rate (MSDR) in the FL as a marker of FL pressurization and found significant associations between MSDR and aortic growth rate.

Despite the intuitive nature of FLEF and MSDR, these parameters are indirect methods for estimating FL pressure. However, recent technical advancements in physics-based image analysis have allowed for non-invasive measurement of intravascular pressure drop from 4D Flow data. *v*WERP (virtual Work-Energy Relative Pressure) is such a technique, which uses a virtual work-energy formulation of the Navier–Stokes equations to provide accurate estimates of relative pressure through complex vascular anatomies [25]. *v*WERP has even been shown to provide accurate measurements (errors of < 1 mmHg) of relative pressure in the TL and FL in-silico, and has been validated against invasive catheterization in-vivo [25]. *v*WERP is thus a promising utility for quantifying intravascular pressure changes in TBAD.

The objective of this study was twofold. First, we aimed to establish the correlation between three techniques for non-invasive estimation of FL pressurization (FLEF, MSDR, and relative pressure changes derived by *v*WERP), and their relation to anatomic risk factors in TBAD. Secondly, we sought to better understand each method's potential applicability for patient risk-stratification by examining associations between aortic growth rate and metrics of FL pressurization in TBAD patients.

## Methods

### Patient identification and clinical/anatomic characteristics

Between November 2014 and August 2019, 22 adult patients with medically managed descending thoracic aorta dissection (n = 19 type B; n = 3 repaired type A) were prospectively enrolled in an IRB-approved study (HUM00120679) and underwent a single research CMR. Patients who underwent research CMR were excluded from analysis for the following reasons: complete FL thrombosis (n = 1), entry tear in the abdominal aorta (n = 1), arrhythmia-related artifact (n = 3), non-contrast CMR (n = 3) or incomplete CMR examination due to claustrophobia (n = 2), resulting in 12 patients available for complete analysis (n = 11 type B; n = 1 repaired type A). All patients had involvement of the descending thoracoabdominal aorta with dominant (i.e., largest) entry tears in the thoracic segment. Clinical and demographic information was collected by research questionnaire and chart review. Baseline anatomic data was measured on the clinical computed tomography (CT) scan acquired at the time of dissection using standard 3D software (Vitrea version 6.9, Vital Images, Toshiba, Tokyo, Japan).

### CMR imaging technique and image analysis

*Acquisition*: All CMR exams were performed on 3 T scanners (n = 1: MR750, General Electric Healthcare, Milwaukee, Wisconsin, USA; n = 11: Ingenia, Philips Healthcare, Best, The Netherlands). The CMR examination included breath-hold, contrast-enhanced magnetic resonance angiography (CE-MRA), using retrospective gating with arrhythmia rejection, and reconstructed at 0.9 mm isotropic resolution after the administration of an iron-based contrast agent (ferumoxytol, 3 mg/kg) in 7 patients at 3 mg/kg dose or gadobenate dimeglumine (Multihance ®; Bracco, Milano, Italy) in 5 patients at 0.2 mL/kg dose. Following the CE-MRA, 4D Flow CMR was performed covering the thoracic aorta. Briefly, 4D Flow scan parameters included: flip angle = 15 degrees, reconstructed resolution = approximately 1.5 × 1.5 × 2.5 mm, acceleration factor = 2.0 × 2.0, views-per-segment = 3, average scan time = 11 min, and average temporal resolution = 47 ms, velocity encoding value = 200 cm/sec. Patient blood pressure was measured by brachial cuff, immediately before commencing scanning, while laying on the scanner table outside of the bore.

**Fig. 1:**
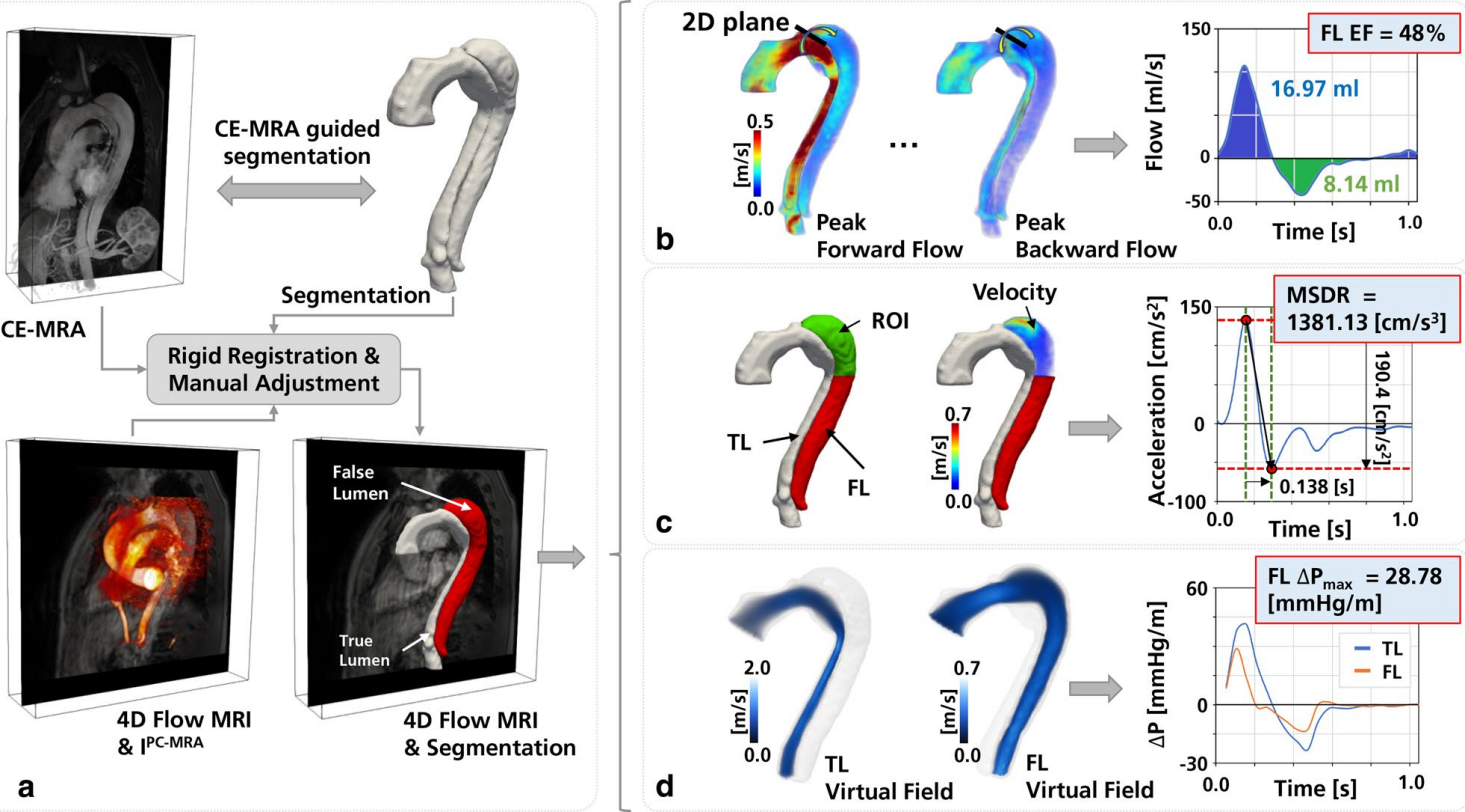
**a** Overview of the cardiovascular magnetic resonance (CMR) post-processing including contrast-enhanced magnetic resonance angiography (CE-MRA) segmentation of the true lumen (TL) and the false lumen (FL) and registration with 4D Flow CMR data. Subsequently, three different flow-based metrics relating to FL pressurization are derived: **b** False lumen ejection fraction (FLEF), calculated as the ratio between retrograde and antegrade flow through the dissection entry tear; **c** Maximum systolic deceleration rate (MSDR), calculated as the absolute difference between peak systolic acceleration and peak systolic deceleration in the TL; **d** Maximal and minimum relative pressures (ΔP_max_ & ΔP_min_), or the difference in absolute pressure between the aortic root and level of the diaphragm, computed for both TL and FL using the image-based virtual Work-energy relative pressure (*v*WERP) approach

*CMR post-processing*: The true lumen (TL) and FL segmentations were generated with dedicated software (Mimics, Materialise, Leuven, Belgium) on CE-MRA images, using contrast thresholding and manual refinement. Once the segmentations were completed, an inhouse MATLAB (MathWorks, Natick, Massachusetts, USA) toolbox [26, 27] was utilized to perform a range of image processing steps: (a) CE-MRA images and 4D Flow CMR were co-aligned using the transformation matrices obtained by the DICOMs tags of both images. (b) CE-MRA images and corresponding segmentations were resampled into the 4D Flow CMR coordinate frame, using linear interpolation. (c) Rigid registration was performed to refine alignment of the TL and FL segmentations with the 4D Flow CMR data. Registration quality was visually assessed by confirming overlap between TL segmentations and the TL time-averaged phase contrast MRA (PC-MRA). In cases where registrations were flagged as in need of further improvement, segmentations were manually adjusted. (d) Lastly, TL and FL segmentations were refined to remove erroneous static tissue voxels at the luminal wall. Once complete, all images were saved for subsequent hemodynamic assessment. A simplified summary of the post-processing of the input data is shown in Fig. 1a.

*False lumen ejection fraction*: 4D Flow CMR DICOM data was uploaded to a web-based software application (Arterys, San Francisco, California, USA) for data reconstruction, visualization and flow analysis. Using this software, FLEF was calculated from the 4D Flow CMR data by performing flow analysis in the plane of the dominant entry tear and using a region of interest that limits measurements to flow through the tear. FLEF was defined as the ratio of retrograde flow volume at the tear during diastole over the antegrade flow volume. The FLEF quantification process is shown in Fig. 1b [18].

*Maximum systolic flow deceleration rate*: To calculate MSDR, a subsection of the FL positioned between the proximal tear and the pulmonary bifurcation, was extracted from the 4D Flow CMR data as described by Ruiz Munoz et al. [24]. In cases where the tear was located at the mid descending aorta, a subsection of 5 cm in length was created around the entry tear. From each subsection, acceleration data was extracted by calculating the time derivative of the mean velocity magnitude in the selected subsection. Using an averaged acceleration trace, MSDR was calculated as the difference between maximum and minimum acceleration during systole, divided by the corresponding time interval between these two points, generating a measure of acceleration or deceleration rate. The MSDR quantification process is shown in Fig. 1c.

*vWERP relative pressure analysis*: *v*WERP was used to assess the development of intraaortic pressure through the TL and FL [25]. The registered segmentations of TL and FL were used as input, with both segmentations connected to the proximal undissected ascending aorta. Relative aortic pressure changes were computed for the TL and FL over a segment from the ascending aorta to the distal FL at the level of the diaphragm (see Additional file 1: Appendix A and Marlevi et al. [25] for technical method details). To account for potential differences in intersubject anatomy, relative pressure traces were normalized by aortic length. From each relative pressure trace, maximum (ΔP_max_) and minimum relative pressure (ΔP_min_) was derived for the TL and FL. Additionally, the transseptal pressure difference was computed by subtracting TL and FL relative pressure. These relative pressures (i.e., TL, FL and transseptal) were chosen for analysis, with positive and negative relative pressures quantifying the acceleration and deceleration of flow through TL and FL, and transseptal pressure representing the force exerted on the dissection septum/flap. The *v*WERP quantification process is shown in Fig. 1d.

*Outcome*: The outcome variable in this study was aortic growth rate, calculated as the change in maximal aortic diameter between baseline computed tomography (CT) angiography and the CE-MRA, measured at the location of maximal aortic diameter of the dissected descending thoracoabdominal aorta, divided by the time interval between scans. Maximal diameter measurements were performed by a reviewer with over 10 years of experience with aortic imaging (N.B.), in a side-by-side fashion using double-oblique and inner-wall to inner-wall measurement technique as this approach has been shown to yield excellent inter-modality measurement agreement [28]. Subjects were categorized as “stable” if their aortic growth rate was < 3 mm/year and “enlarging” if the rate was ≥ 3 mm/year [29].

*Reproducibility analysis*: To assess the reproducibility of the FLEF measurements, a second independent rater with 4 years experience with aortic imaging, who was blinded to aortic growth data, performed manual definition of the entry tear plane and subsequent FLEF measurements on the TBAD 4D Flow datasets. Subsequently, once FLEF re-measurements were finalized, the same reviewer performed re-measurement of maximal aortic dimensions at baseline and follow-up CTs with subsequent determination of aortic growth.

For MSDR and FL ΔP_max_, the CE-MRA and 4D Flow CMR datasets were re-aligned using the same procedure described in CMR *post-*processing above, and subsequently, three sets of alterations were introduced to represent different modes of segmentation variations: one being systematically smaller than the original segmentations (representing the instance of threshold segmentation using a more conservative threshold value), one being systematically larger than the original segmentations (representing the instance of threshold segmentation using a more inclusive threshold value), and one being interchangeable smaller or larger with alterations varying along the length of the aorta (representing the instance of manual segmentation). For all sets, MSDR and FL ΔP_max_ were re-evaluated (for details on the reproducibility analysis, see Additional file 1: Appendix B).

Measurement reproducibility was assessed using Pearson’s correlations, limits-of-agreement (LOA) statistics and Bland–Altman plots.

### Statistics

Patient characteristics are reported as mean ± SD for normally distributed continuous variables, medial and interquartile range (IQR) for non-normal continuous variables, and frequencies for categorical variables. Normality was assessed using the Shapiro–Wilk test. Pearson’s correlation was used to determine associations between aortic growth rate, anatomic and hemodynamic parameters. Comparison of group means for continuous variables was performed with unpaired t-tests or Mann–Whitney U test. Chi-square analysis and Fisher’s exact test were used to evaluate differences in frequency of categorical variables. Pairwise correlation matrices were used to identify multicollinearity amongst predictors. Subsequently, multiple linear regression models with robust standard errors were used to examine the association of hemodynamic metrics with aortic growth after adjusting for baseline aortic diameter. A *p*-value of < 0.05 was considered significant for all statistical tests. Statistical analyses were performed using Stata (version 14.0, StataCorp LP, College Station, Texas, USA).

## Results

### Patient characteristics

Patient and anatomic characteristics are reported in detail Table 1. The average patient age was 54.9 ± 9.6 years (range: 31–71 years), with a majority of the patients being male (75%). A majority of the patients had a history of hypertension (83%), whereas only a minority had an established history of connective tissue disease (25%), and frequencies of these variables did not significantly differ between stable and enlarging groups. Mean systolic and diastolic blood pressure were 130 ± 18 (range: 98–152 mmHg) and 70 ± 13 mmHg (range: 49–89 mmHg), respectively, and the mean heart rate was 57 ± 8 bpm (range: 47–75 bpm). All patients were receiving beta-blockers for medical management, and at the time of CMR the majority of patients (8/12) were considered to be achieving a blood pressure goal of < 140/90 mmHg; among the 4 patients who were not at blood pressure goal, 3 were in the enlarging group and 1 was in the stable group. The average age of the dissection at the time of CMR was 3.6 ± 3.3 years. Patients in the stable group had a significantly longer history of dissection (7.8 ± 0.3 vs. 1.5 ± 1.5 years for the enlarging group, p < 0.001).

**Table 1:** Patient characteristics and demographics

Characteristics	Overall (n = 12)	Stable (n = 4)	Enlarging (n = 8)	p-value
Patient age (years)	54.9 ± 9.6 (range: 31–71)	47.8 ± 11.5	58.5 ± 6.6	0.157
Sex (male/female), n	9/3	3/1	6/2	1.000
Hypertension, n (%)	10 (83)	2 (50)	8 (100)	0.091
Smoking history, n (%)	6 (50)	1 (25)	5 (63)	0.545
History of connective tissue disease, n (%)	3 (25)	2 (50)	1 (12)	0.236
Age of dissection at CMR (years)	3.6 ± 3.3 (range: 0.2–8.0)	7.8 ± 0.3	1.5 ± 1.5	< 0.001
Maximum diameter at baseline (mm)	41.3 ± 8.4 (range: 29–58)	38.0 ± 6.1	42.9 ± 9.2	0.302
Maximum diameter at CMR (mm)	48.8 ± 8.9 (range: 32–62)	42.3 ± 10.4	51.8 ± 6.8	0.187
Aortic growth rate (mm/year)*	6.1 (1.3–11.0) (range: 0–21.7)	0.6 (0–2.0)	11.1 (3.3–21.7)	0.004
Thoracic aortic dissection length (cm)	25.9 ± 3.6 (range: 18.4–32.4)	27.4 ± 4.1	25.1 ± 3.3	0.364
Entry tear distance from left subclavian artery (mm)*	20.0 (7.5–31) (range: 0–130)	20.0 (7.5–76)	20.0 (9.5–31)	0.932
Dominant entry tear size by CMR (mm)*	17.8 (14.5–21.3) (range: 10.8–44.5)	24.3 (14.2–37.8)	17.6 (14.5–20.3)	0.500
Systolic blood pressure (mmHg)	130 ± 18 (range: 98–152)	122 ± 16	134 ± 19	0.307
Diastolic blood pressure (mmHg)	70 ± 13 (range: 49–89)	70 ± 12	70 ± 14	1.000
Pulse pressure by CMR (mmHg)	60 ± 11 (range: 44–80)	52 ± 8	64 ± 11	0.070
Heart rate (bpm)	57 ± 8 (range: 47–75)	57 ± 9	57 ± 8	0.931

^*^Median (IQR)

Regarding anatomical variables, the maximum baseline aortic diameter (based on index CT) was 41.3 ± 8.4 mm (range: 29–58 mm), increasing to 48.8 ± 8.9 mm (range: 32–62 mm) at the time of CMR, and the median aortic growth rate between baseline CT and CMR was 6.1 mm/year (IQR: 1.3–11.0; range: 0–21.7 mm/year). The mean entry tear size was 17.8 mm (IQR: 14.5–21.3 mm), and the distance of the entry tear from the left subclavian artery was 20.0 mm (IQR: 7.5–31.0 mm). As would be expected, aortic growth rate was significantly higher in the enlarging subgroup (11.1 ± 7.0 mm/year vs. 0.6 ± 0.9 mm/year, p = 0.004). Anatomical variables including dissection length, entry tear size, distance of the entry tear from the left subclavian artery and baseline maximal diameter were similar between groups (p = NS). Note that for the anatomical magnitude images, difference in signal-to-noise (SNR) was inferred between the two utilized contrast agents (21.5 ± 5.7 vs. 55.9 ± 18.5, p = 0.003, for ferumoxytol vs. multihance), with SNR calculated as the mean velocity in the entire segmented aorta divided by the standard deviation of the background noise, identified in a region outside the thoracic cavity [30].

### Hemodynamic assessment

Mean cardiac output was 4.9 ± 0.9 l/min, mean ascending aortic forward flow was 87.4 ± 21.7 ml/beat and mean ascending aortic reverse flow was − 7.7 ± 7.5 ml/beat; these parameters did not differ between stable and enlarging groups.

**Fig. 2:**
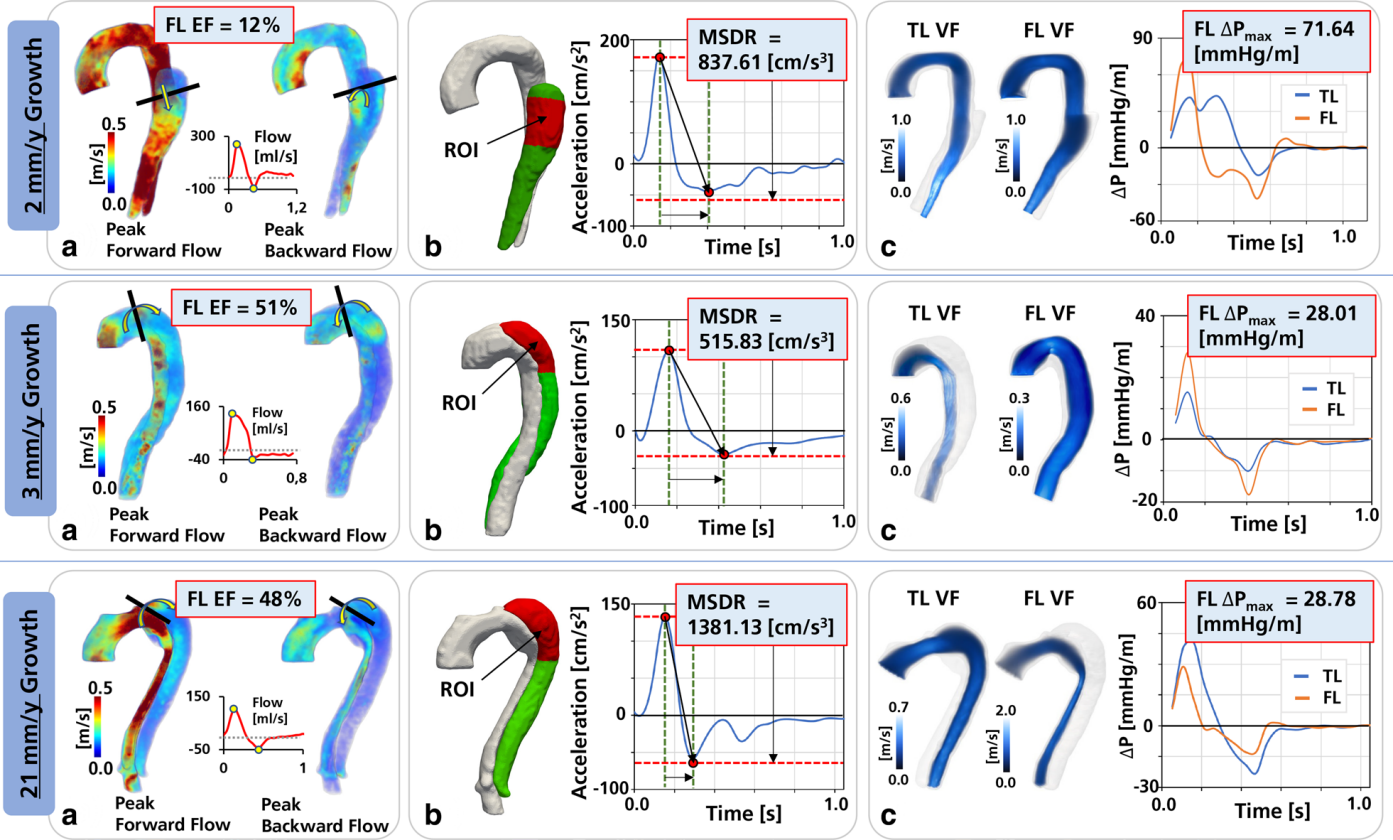
Representative patient examples of FL hemodynamic evaluation, provided for two subjects with slow aortic growth (top and middle row), and one with rapid aortic growth (bottom row). In all instances, extraction of FLEF (**a**), MSDR (**b**), and relative pressure by *v*WERP (**c**) is shown together with associated output (VF = virtual field)

The average net flow rate was higher in the TL than in the FL (3.4 ± 1.1 vs. 1.5 ± 1.4 l/min, p = 0.004). Peak velocity of the entry tear jet was similar between stable and enlarging groups (108.1 ± 11.7 vs. 99.2 ± 8.7 cm/s, p = 0.56). Overall mean FLEF was 36.0 ± 24.7% (range 0 to 88%) and FLEF was significantly higher in enlarging vs stable aortic dimensions (49.0 ± 17.9% vs 10.0 ± 11.9%, p = 0.002). In comparison, overall mean MSDR was 1401 ± 956 cm/s^3^ and did not differ significantly between groups (1146 ± 782 vs. 1529 ± 1058 cm/s^3^, p = 0.499). For the assessment of intra-aortic pressure development, a significant decrease in FL ΔP_max_ was observed in patients with enlarging vs stable aortic dimensions (32.3 ± 10.8 vs. 57.2 ± 12.5 mmHg/m, p = 0.017); however, no difference was observed between FL ΔP_min_ (− 17.6 ± 6.6 vs. -28.7 ± 9.8 mmHg/m, p = 0.105). Two subjects (one with stable, and one with enlarging aortic growth) had TL values excluded from analysis due to severe TL narrowing in the descending aorta leading to data loss (average TL radius < 2 image voxels). Among the remaining analyzed patients, no significant differences in TL maximum relative pressure (46.6 ± 7.5 vs 51.9 ± 11.6 mmHg/m, p = 0.418), and TL minimum relative pressure (− 23.6 ± 5.7 vs. − 21.6 ± 6.1 mmHg/m, p = 0.654) were observed between stable and enlarging groups. Table 2 provides a summary of all evaluated hemodynamic parameters. Representative examples of FL hemodynamic evaluation for patients with varying degrees of aortic growth are shown in Fig. 2. Note that no difference in velocity-to-noise (VNR) could be inferred in the 4D Flow CMR data as a function of the two different contrast agents (32.5 ± 13.0 vs. 41.5 ± 17.6, p = 0.209, for ferumoxytol vs. gadolinium). Here, VNR was calculated using the relationship given in Beerbaum et al. [30], being directly proportional to the velocity encoding divided by the standard deviation of velocity noise in the static tissue (extracted from a region of interest positioned in the posterior back muscles).

**Table 2:** Hemodynamics parameters derived from 4D Flow CMR

Characteristics	Overall (n = 12)	Stable (n = 4)	Enlarging (n = 8)	p-value
*Ascending aorta*				
Cardiac Output (l/min)	4.9 ± 0.9 (range 3.3–6.4)	5.1 ± 0.9	4.8 ± 1.0	0.621
Forward Flow (ml/beat)	87.4 ± 21.7 (range 58.2–125.2)	90.9 ± 24.7	85.7 ± 21.6	0.734
Reverse Flow (ml/beat)	− 7.7 ± 7.5 (range − 26.8−0.5)	− 5.2 ± 3.2	− 8.9 ± 8.9	0.314
*True lumen*				
Net flow (l/min)	3.4 ± 1.1 (range 2.1–5.5)	3.0 ± 1.0	3.6 ± 1.1	0.474
Peak velocity (cm/s)	89 ± 32 (range 38–166)	84 ± 7	91 ± 40	0.653
Maximum relative pressure (mmHg/m)*	50 ± 11 (range 41–75)	47 ± 8	52 ± 12	0.418
Minimum relative pressure (mmHg/m)*	− 22 ± 6 (range − 32 to − 15)	− 24 ± 6	− 22 ± 6	0.654
*False lumen*				
Net flow (l/min)	1.5 ± 1.4 (range 0.1–4.6)	1.9 ± 2.2	1.2 ± 0.9	0.596
Peak velocity at entry tear (cm/s)	102 ± 10 (range 68–144)	108 ± 12	99 ± 9	0.560
Peak velocity (cm/s)	68 ± 31 (range 16–120)	67 ± 40	69 ± 29	0.925
False lumen ejection fraction (%)	36 ± 25 (range 0–88)	10 ± 12	49 ± 18	0.002
Maximum systolic deceleration rate (cm/s^3^)	1401 ± 956 (range 516–3201)	1146 ± 782	1529 ± 1058	0.499
Maximum relative pressure (mmHg/m)	41 ± 16 (range 21–72)	57 ± 13	32 ± 11	0.017
Minimum relative pressure (mmHg/m)	− 21 ± 9 (range − 42 to − 11)	− 29 ± 10	− 18 ± 7	0.105
*Transseptal*				
Maximum relative pressure (mmHg/m)*	35 ± 23 (range 1–66)	25 ± 36	39 ± 18	0.585
Minimum relative pressure (mmHg/m)*	− 14 ± 11 (range − 34 to − 4)	− 14 ± 18	− 14 ± 10	0.989

For characteristics marked *, subjects were excluded due to excessive TL narrowing

### Correlation analyses

None of the baseline anatomic or demographic variables such as maximum aortic baseline diameter (r = 0.45, p = 0.143), entry tear size (r = − 0.21, p = 0.509), entry tear distance from the left subclavian artery (r = 0.22, p = 0.497), dissection length (r = 0.23, p = 0.466), systolic blood pressure (r = 0.11, p = 0.745), heart rate (r = − 0.20, p = 0.533) or pulse pressure (r = 0.50, p = 0.100) were significantly correlated with aortic growth rate on bivariate analysis. Only one correlation between anatomic and hemodynamic parameters was statistically significant: entry tear distance from left subclavian artery vs. FL peak velocity (− 0.72, p = 0.009).

**Fig. 3:**
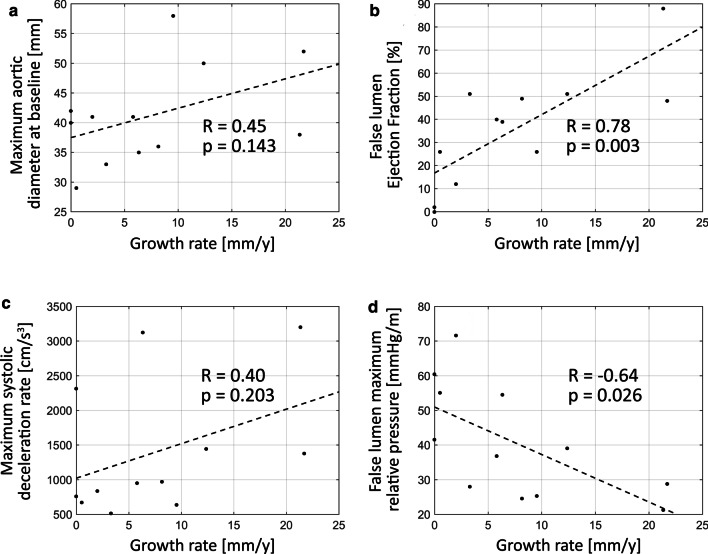
Scatter plots depicting the correlation between aortic growth rate and **a** baseline maximal aortic diameter, **b** false lumen ejection fraction, **c** maximum systolic deceleration rate, and **d** false lumen maximum relative pressure

Among the three investigational metrics, FLEF showed a strong positive correlation with growth rate (r = 0.78, p = 0.003), whereas FL ΔP_max_ showed a moderate-strong negative correlation with growth rate (r = − 0.64, p = 0.026). FLEF and FL ΔP_max_ also demonstrated a moderate-strong correlation with each other (r = − 0.67, p = 0.017); however, these quantities did not correlate with any other hemodynamic or anatomic parameters (including entry tear size, tear distance from left subclavian artery, systolic blood pressure, and pulse pressure). FL ΔP_min_ also demonstrated a significant, but slightly weaker correlation with growth rate (r = 0.59, p = 0.043) than FL ΔP_max_, and was strongly correlated with FL ΔP_max_ (r = − 0.96, p < 0.001). MSDR demonstrated a weak-moderate, but non-significant correlation with aortic growth rate (r = 0.40, p = 0.203), and a moderate-strong positive correlation to TL peak velocity (r = 0.64, p = 0.004). MSDR did not correlate with FLEF (r = 0.37, p = 0.24), FL ΔP_max_ (r = − 0.11, p = 0.73) or FL ΔP_min_ (r = 0.12, p = 0.71). Cardiac output and heart rate were not correlated with FLEF, FL ΔP_max_, or MSDR. Scatter plots depicting the correlation between aortic growth rate and key parameters are shown in Fig. 3.

### Multivariate analyses—predictors of aortic growth rate

Multiple linear regression analysis models were performed to identify the independent association of FLEF, FL maximum relative pressure and MSDR on aortic growth rate after adjusting for baseline maximal diameter (three separate multivariate analyses were utilized to avoid multicollinearity). On adjusted analysis, FLEF was independently associated with aortic growth rate (β = 0.23, p < 0.001) with an overall model adjusted R^2^ = 0.85. Similarly, FL ΔP_max_ was also independently associated with aortic growth rate (β = − 0.22, p = 0.012), with an overall model adjusted R^2^ = 0.60. However, MSDR was not found to be independently associated with aortic growth rate after adjusting for baseline diameter (β = − 0.003, p = 0.151). Full data is shown in Table 3.

**Table 3:** Multivariate regression assessment—evaluating independent predictors of aortic growth rate

Characteristics	β coefficient	95% CI	p-value
*Predictors of growth rate with FL EF (adjusted R*^*2*^ = *0.85)*			
FLEF	0.23	0.18, 0.32	< 0.001
Baseline maximum aortic diameter	0.45	0.12, 0.79	0.013
*Predictors of growth rate with FL ΔP*_*max*_* (adjusted R*^*2*^ = *0.60)*			
FL maximum relative pressure	− 0.22	− 0.50, − 0.01	0.012
Baseline maximum aortic diameter	0.27	− 0.27, 0.81	0.283
*Predictors of growth rate with MSDR (adjusted R*^*2*^ = *0.42)*			
MSDR	0.003	− 0.002, 0.009	0.151
Baseline maximum aortic diameter	0.47	0.50, 0.88	0.031

*FL* false lumen, *FLEF* false lumen ejection fraction, *MSDR* maximum systolic deceleration rate

### Reproducibility analysis

Complete results for the reproducibility analysis are provided in Additional file 1: Appendix B. Comparing aortic growth rate assessed by different raters, strong correlation was observed (r = 0.94, p < 0.001). A modest interrater difference of 1.5 ± 2.8 mm/year was noted for aortic growth rate, although without significant bias and with concordant growth categorization (stable vs. enlarging) between raters in all cases (see Additional file 1: Appendix Figure B.1 and Table B.1 for complete data).

The two rater’s measurements of FLEF were also strongly correlated (r = 0.86, p < 0.001), with a mean difference of − 1.6 ± 13.4%. For FL ΔP_max_, strong correlation was observed after repeat mask registration and with additional systematic variations in segmentation (r > 0.94, p < 0.001), with the largest difference observed for masks with systematically dilated segmentations (− 2.7 ± 9.2 mmHg/year). For MSDR, moderate-strong correlation also persisted over all variations in segmentation (r > 0.75, p < 0.005), with the largest difference observed for systematically eroded segmentations (− 133 ± 685) (complete data presented in Additional file 1: Appendix B).

## Discussion

In this study, we examined the ability of three 4D Flow CMR derived methods of estimating FL pressurization to predict growth in patients with chronic dissection of the descending thoracic aorta: FLEF, MSDR, and *v*WERP-derived FL maximum relative pressure (FL ΔP_max_). We have shown that FL hemodynamics are significantly altered in patients with aortic growth: FLEF was significantly increased and FL ΔP_max_ was significantly decreased. Neither MSDR, nor any of the anatomical metrics (such as baseline maximum aortic diameter) clearly differentiated enlarging from stable patients. TL and transseptal hemodynamic parameters did not differ between groups, which is unsurprising considering aortic growth in TBAD is the direct consequence of FL enlargement. Furthermore, FLEF and FL ΔP_max_ demonstrated moderate-strong correlation with each other, and both were found to be independent predictors of aortic growth after adjusting for baseline aortic diameter. This work is the first application of *v*WERP to provide a direct pressure assessment of the FL in TBAD patients, and to understand the relation between direct and indirect techniques for assessing FL pressure using 4D Flow CMR. These results lend further credence to the importance of FL pressurization in promoting growth in TBAD, and support further investigation of FLEF and FL ΔP_max_ as hemodynamic biomarkers of risk in TBAD patients.

### Current approaches and mechanisms of aortic growth

Current treatment protocols and surgical criteria are largely based on anatomic variables, the most important of which is maximum aortic diameter [7]. Aortic diameter is a simple metric, and has a direct relationship with tensile wall stress (i.e., Law of Laplace). However, in a recent systematic review of growth in TBAD, maximal aortic diameter was associated with growth in only ~ 50% of studies, highlighting the need for better risk stratification tools [7]. While our results support the association between baseline diameter and aortic growth we also found that FLEF and FL ΔP_max_ provided additional predictive value over baseline diameter alone. This is likely due to the fact that anatomic variables such as aortic diameter are a consequence of aortic wall pathology rather than a direct cause of it. While aortic growth is the result of a complex set of factors (e.g., hemodynamic stress, mechanobiological responses, aortic tissue strength), most of these factors are challenging to non-invasively measure in patients, highlighting the unique value of hemodynamic assessment by 4D Flow CMR.

### Metrics of false lumen pressurization

In this study, we chose to assess the predictive value of three different methods of assessing FL hemodynamic stress: FLEF, MSDR, and FL ΔP_max_. These three metrics represent different ways of—either directly or indirectly—describing the relationship between flow and pressure in the FL, and the three also represent different ways of interrogating the acquired flow field: utilizing bulk flow, acceleration rate, or relative pressure, respectively.

**Fig. 4:**
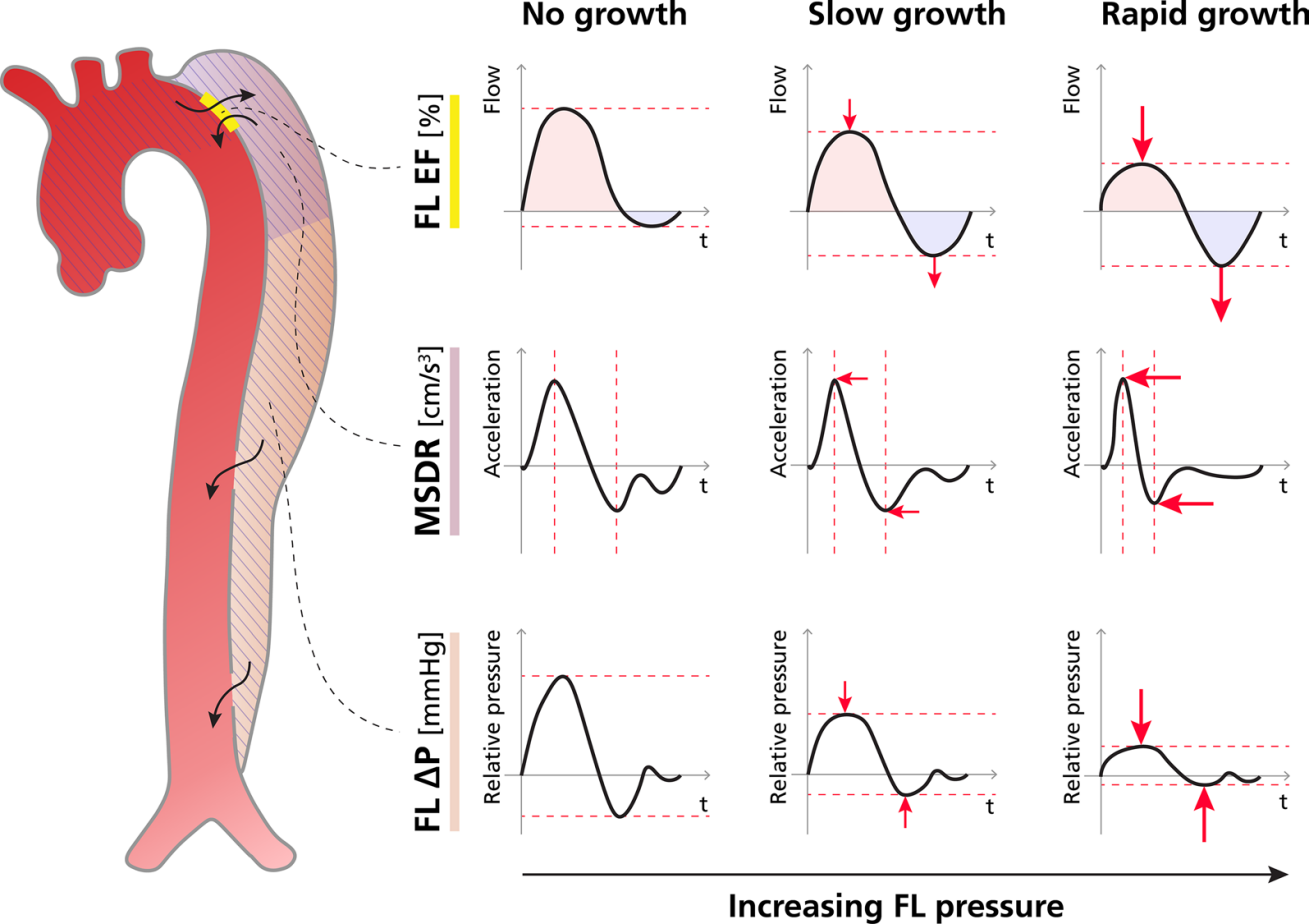
Conceptual model depicting the proposed relations between growth and 4D Flow-derived markers of FL pressurization. False lumen ejection fraction (FLEF): The top row depicts increased retrograde flow (light blue) relative to antegrade flow (light red) with increasing aortic growth rate, hypothesized to be related to increased FL pressurization. Maximum systolic deceleration rate (MSDR): The middle row depicts the acceleration of blood through a proximal portion of the FL (the light purple shaded area), with MSDR representing the mean rate of change between peak acceleration and peak deceleration (i.e. the downward slope between peaks). With increasing FL pressurization, higher resistance FL flow leads to faster flow deceleration (i.e. a more pronounced, steeper slope). FL maximal relative pressure (FL ΔP_max_): The bottom row depicts the observed trend between decreased relative pressure between the aortic root and the distal FL (the striped region in the TBAD to the left) and increasing aortic growth rate. Increased FL pressurization leads to increased resistance to flow, and thus dampening of relative pressure gradients

FLEF has been previously described in TBAD [18], with this metric describing the ratio between retrograde and antegrade flow through the dominant entry tear. FLEF is a regional and indirect measure of assessing FL pressurization. An increasing proportion of FL inflow relative to outflow will result in increased FL pressure (particularly diastolic pressure [12]) and increased resistance to forward flow. During diastole, flow reversal occurs when FL diastolic pressure supersedes diastolic pressure in the TL, and this reversed pressure gradient drives blood from the FL into the TL across entry tears. This ratio of retrograde and antegrade flow at the entry tear, mediated by diastolic pressure gradients, can thus be posed as a surrogate measure of the pressure difference between FL and TL (a conceptual illustration of the relationship between antegrade/retrograde and aortic growth is show in Fig. 4). Interestingly, the relationship between retrograde flow and pressure overload has been described not only in the FL of TBAD patients, but also as a maker of pulmonary hypertension severity [31]. Advantages to the FLEF approach include: bulk flow rate measurements in TBAD with 4D Flow CMR have been shown to be highly reproducible [32] and are easily performed using a variety of commercially available software, flow measurements are performed at a discreet anatomic location (i.e., entry tear) and are thus fairly robust to variations in dissection anatomy, and this approach avoids technical difficulty and potential inaccuracy related to conversion of spatiotemporal flow gradients in its computation. However, FLEF does not directly measure pressure, does not take into account the hemodynamics at distal re-entry tears, and the definition of a 2-dimensional flow analysis plane can be difficult if entry tear anatomy is complex. Nevertheless, the moderate-strong correlation between FLEF and aortic growth in our data underlines this metrics potential clinical utility.

Alternatively, MSDR is a recently proposed semi-regional, indirect method of assessing FL pressurization [24], representing the average peak systolic deceleration rate in a proximal sub-section of the FL. As with FLEF, the coupling to FL pressurization is intuitive: with increasing FL pressures, resistance to forward flow will increase, antegrade systolic flow will decelerate more rapidly, and the MSDR will consequently increase (see Fig. 4 for a conceptual depiction of changes in MSDR with increasing aortic growth). MSDR was recently introduced and studied in a cohort of 29 patients (combined repaired type A dissection and TBAD), where a weak-moderate, positive correlation with aortic growth rate (r = 0.48) was reported [24]. While we identified a similarly weak-moderate positive correlation between MSDR and aortic growth rate (r = 0.40), this correlation did not reach statistical significance, possibly owing to the smaller size of our cohort. However, the weaker correlation between MSRD and aortic growth compared to FLEF and FL ΔP_max_ may be a result of the MSDR metric itself, given that the MSDR measurement relies on SNR and the temporal sampling rate of the acquired flow field, which can be variable in TBAD. Furthermore, the presence of significant secondary flow features (e.g., helices and vortices) may affect MSDR measurements, and appropriate definition of the analysis subsection becomes less clear when the entry tear is located more distally along the descending aorta.

While FLEF and MSDR are proposed as indirect measures of FL pressure, recent developments in physics-based image analysis now enable the accurate extraction of relative pressure over vascular sections. *v*WERP is a validated method for relative pressure measurement that utilizes the concept of virtual work-energy, which has shown particular promise in assessing complex vasculatures and has been explicitly tested in TBAD anatomy [25]. As such, the FL ΔP_max_ represents a global and direct measure of change in pressure from the ascending aorta to the distal thoracic FL. FL ΔP_max_ should thus decrease with increasing FL pressure (and constant ascending pressure) and growth. This is concordant with our observed moderate-strong negative correlation between FL ΔP_max_ and aortic growth rate (see Fig. 4 for a conceptual depiction of changes in FL relative pressure with increasing aortic growth). Similar to FLEF and MSDR, *v*WERP can be derived from 4D Flow data alone, however, the method does require the creation of an auxiliary virtual field in order to compute relative pressure. Although not an overly time-consuming process (~ 5 min on a local desktop computer), *v*WERP computation requires aortic segmentation, which can be time-consuming in TBAD, and separate computational implementation, making it a more complex analysis than other measures such as FLEF. Lastly, an advantage of the vWERP approach is that relative pressure is derived over the entire thoracic aorta, and thus the global hemodynamic state of the FL is accounted for in its computation.

When considering the differences between FLEF and FL ΔP_max_, we found a slightly stronger correlation of FLEF with aortic growth compared to FL ΔP_max_ on both adjusted and unadjusted analyses (r = 0.78 vs. r = − 0.64, Fig. 3), although it's unclear if such small differences in strength of correlation are meaningful or simply related to statistical noise. The simplicity in derivation makes FLEF an attractive metric in clinical instances where the FL has a single dominant tear in the thoracic aorta, whereas *v*WERP may be better suited in scenarios with multiple or complex flap fenestrations/tears (as shown in previous in-silico work [25]. Regardless of the metric, our analysis underlines the pathophysiological importance of FL pressurization in TBAD growth, adding to the increasing number of studies highlighting its diagnostic role [10–13, 18, 24, 33]. In reality, since both FLEF and FL ΔP_max_ can be measured from the same 4D Flow acquisition, measurement of multiple parameters may be a complementary approach that lends additional diagnostic certainty in cases where pressurization assessments are concordant.

Lastly, it is important to acknowledge potential sources of measurement variability in these hemodynamic metrics: manual plane placement at the entry tear for FLEF, and aortic segmentation for MSDR and FL ΔP_max_. The results from our reproducibility analyses address this in detail (Additional file 1: Appendix B). In brief, for the three derived hemodynamic metrics we identified no significant inter-rater bias, although agreement was highest for FL ΔP_max_ and lowest for FLEF. However, the degree of variability for FLEF and FL ΔP_max_ were substantially lower than the mean differences in these metrics between stable and enlarging groups, suggesting that this degree of variability is still acceptable for separating patients at high and lower risk of growth. Additionally, FL ΔP_max_ demonstrated higher variability related to aortic mask dilation rather than erosion into the lumen, a behavior that is consistent with prior *v*WERP results [34]. Along the same lines, modest interrater differences were noted with measurement of aortic growth rate, an observation which is not surprising given the known variability of diameter measurement in dissected aortas; however, growth categorizations (stable vs. enlarging) remained concordant between readers and for all cases despite this measurement variability. Nevertheless, care must be taken when deriving any of the aforementioned metrics in future scientific or clinical studies, and raters with experience in accurately delineating dissection anatomy are key for reliable anatomic or hemodynamic assessment.

### Limitations

Our study has several limitations. First, our study cohort was relatively small in size, and thus these findings should be interpreted as preliminary and hypothesis generating, although efforts to study these metrics in larger cohorts are ongoing. In part our small sample size was due to the fact that we excluded 3 patients for non-contrast exams (inability to accurately segment the TL and FL) and another 3 patients for arrhythmia-related artifacts. However, we believe that these results can be viewed as a representative example of how advanced flow imaging can provide unique insights into the complex hemodynamic mechanism of TBAD.

Second, exclusively chronic dissections were analyzed in our study, and as such it is impossible to infer causal relationships between aortic growth and our three investigational metrics. However, given that abnormal FL blood flow been linked to aneurysmal growth in previous imaging studies of acute TBAD patients [8] and in computational studies [35], we believe it is reasonable to assume that such abnormal hemodynamics are a precursor of progressive FL growth. Efforts are ongoing to validate these findings in acute/subacute TBAD patients.

Third, although not unique to our study, the acquisition of 4D Flow CMR data is not available or part of routine clinical CMR protocols at most centers, although such data can be acquired on almost all modern clinical CMR systems. Further investigation into the specific clinical utility of these metrics of FL pressurization will be needed to promote more wide-spread clinical translation. Lastly, aortic growth was determined by measuring diameter changes between different modalities (e.g., CT at baseline and MRA at follow-up). However, a recent study comparing inter-modality differences in aortic measurements indicate only small and non-significant differences when a consistent measurement technique is used [28], as was done in this study.

## Conclusions

Using non-invasive 4D Flow CMR, three different metrics of FL pressurization were evaluated in conjunction with aortic dissection growth: FLEF, MSDR, and FL ΔP_max_. FLEF and FL ΔP_max_ were correlated with each other, and both differentiated patients with stable vs. enlarging aortic dimensions and both were predictive of aortic growth rate after adjustment for baseline aortic diameter. Conversely, MSDR did not significantly correlate with aortic growth or other investigational hemodynamic metrics. Overall, these results highlight the possible clinical value of non-invasive FL pressurization assessment in patients with aortic dissection of the descending aorta, and highlight the potential role of 4D Flow CMR in providing patient-specific hemodynamic assessments for improved patient management.

## Supplementary Information

**Additional file 1.** Additional material - Appendix A (Relative pressure estimation by vWERP) and B (Reproducibility analysis).

## Data Availability

The datasets used and/or analyzed during the current study contain patient data, but can access can be requested from the corresponding author on reasonable request and after appropriate agreements.

## Abbreviations

Δp_max_: Relative pressure
CE-MRA: Contrast enhanced magnetic resonance angiography
CI: Confidence interval
CMR: Cardiovascular magnetic resonance
CT: Computed tomography
FL: False lumen
FLEF: False lumen ejection fraction
IQR: Interquartile range
MSDR: Maximum systolic deceleration rate
PC- MRA: Phase contrast magnetic resonance angiography
SD: Standard deviation
TBAD: Type B aortic dissection
TEVAR: Thoracic aortic endovascular repair
TL: True lumen
VNR: Velocity to noise
*v*WERP: Virtual work-energy relative pressure
