# Supplementary material for: False lumen pressure estimation in type B aortic dissection using 4D flow cardiovascular magnetic resonance: comparisons with aortic growth
Source: J Cardiovasc Magn Reson. 2021 May 13;23:51. doi: 10.1186/s12968-021-00741-4 (PMC8117268; doi:10.1186/s12968-021-00741-4)
Supplement: Supplementary file 1 — Additional file 1. Additional material - Appendix A (Relative pressure estimation by vWERP) and B (Reproducibility analysis). [file 12968_2021_741_MOESM1_ESM.docx]

**ADDITIONAL MATERIAL**

**APPENDIX A – Relative pressure estimation by *v*WERP**

The virtual Work-Energy Relative Pressure (*v*WERP) method - utilized to estimate changes in pressure over arbitrary vascular regions - originates from the Navier Stokes equations, defining the relationship between fluid velocity and pressure as

| $\rho\frac{\partial}{\partial t}\boldsymbol{v+}\rho\boldsymbol{v}\cdot\nabla\boldsymbol{v}-\mu\nabla^{2}\boldsymbol{v}+\nabla p=0$ | (1) |
| --- | --- |
| $\nabla\cdot\boldsymbol{v}=0$ | (2) |

with $\boldsymbol{v}$ being velocity, $p$ pressure, $\rho$ fluid density, and $\mu$ dynamic viscosity. As derived in previous work (25), a *virtual* work-energy form can then be reached by multiplying Eq. (1) with an introduced, auxiliary virtual field $\boldsymbol{w}$, and evaluating each resulting term over the vascular section of interest $\Omega$ with inlet plane $\Gamma_{i}$ and outlet plane $\Gamma_{o}$. The final *v*WERP form for estimating relative pressure $\Delta p$ then takes on the form

| $\Delta p= -\frac{1}{Q_{e}}\left( \frac{\partial}{\partial t}K_{e}+A_{e}+V_{e} \right)$ | (3) |
| --- | --- |

with each entry in (3) representing a *virtual* energy component relating to both $\boldsymbol{w}$ and $\boldsymbol{v}$ as

| $\frac{\partial}{\partial t}K_{e}=\rho\int_{\Omega} \frac{\partial\boldsymbol{v}}{\partial t}\cdot\boldsymbol{w}d\Omega$ | (4) |
| --- | --- |
| $A_{e}= \rho\int_{\Omega} \left( \boldsymbol{v}\cdot\nabla\boldsymbol{v} \right)\cdot\boldsymbol{w}d\Omega$ | (5) |
| $V_{e}= \rho\int_{\Omega} \nabla\boldsymbol{v}:\nabla\boldsymbol{w}d\Omega$ | (6) |
| $Q_{e}=\int_{\Gamma_{i}} \boldsymbol{w}\cdot\boldsymbol{n}d\Gamma$ | (7) |

All terms above can be directly derived from acquired 4D Flow MRI data (representing the velocity field $\boldsymbol{v}$), and does not require any additional measurements. The only additional feature needed is a numerical solved $\boldsymbol{w}$, posed in this work as a Stokes flow problem with a plug inflow profile. Full theoretical details, including pre-processing and detailed method output performance can be found in Marlevi et al (25).

**APPENDIX B – Reproducibility analysis**

An interobserver variability analysis was performed to assess the variability of measured aortic growth rate, FL EF, MSDR, and FL ΔP_max_, respectively. For aortic growth rate and FL EF, this was performed by having a second independent rater derive those very same metrics in the original image data (identifying maximum aortic diameter at baseline and follow-up studies to determine growth rate, and delineating the TBAD opening tear for FL EF, respectively). For MSDR and FL ΔP_max_, new sets of segmentations were generated by repeating the semi-automated CE-MRA to 4D Flow MRI mask registration procedure. Additionally, to investigate the sensitivity of MSDR and FL ΔP_max_  to variations in segmentation we systematically altered the registered segmentation masks in a systematic fashion as described below:

In short, alterations in the aortic masks were created based on the notion that two primary modes of segmentation variability exist: one mode is through differences in thresholding of the MRA data, in which would result in either systematically smaller or larger segmentations at the aortic boundary, depending on the utilized threshold value. The second mode would be related to manual editing of segmentations, where random and spatially varying deviations from the aortic boundary could be introduced. To investigate these effects we created 3 new sets of segmentations, after the repeated image registration:

1. Segmentations being systematically *smaller* than the original ones, achieved by eroding the entire segmentation by one layer of voxels
2. Segmentations being systematically *larger* than the original ones, achieved by dilating the entire segmentation by one layer of voxels
3. Segmentations being variably *smaller or larger*, achieved by randomly sampling 80 points along the segmentation wall, then allowing half of them (n=40) to dilate a regional neighborhood (R = 1cm) by 1 voxel, and allowing the other half (n=40) to erode a regional neighborhood (R = 1cm) by 1 voxel.

Here, 1. and 2. represent the first mode of segmentation (thresholding), whereas 3. represents the second mode (manual segmentation). For all three sets, MSDR and FL ΔP_max_ were re-evaluated.

Complete results are shown in **Table B.1**, as well as highlighted in **Figure B.1-B.3**. For aortic growth rate there was a strong correlation between raters measurements (r = 0.94, p<0.001), with a mean difference of 1.46 ± 2.79 mm/year. There was 1 case with a significant discrepancy noted between raters (3.4 vs. 12.3 mm/y) although the classification of stable and enlarging subjects remained concordant between readers and this magnitude of this discrepancy was primarily an effect of the short time interval.

For the multiple readings of FL EF, strong correlation was reported for the entire cohort (r = 0.86, p<0.001), with no significant bias and average variability of -1.6 ± 13.4%).

For FL ΔP_max_, repeat mask registration alone generated small variability of -0.5 ± 4.0 mmHg/m, with the two readings being strongly correlated (r = 0.98, p < 0.001) (see **Figure B.1** and **Table B.1**). When adding systematic segmentation errors (erosion, dilation and random variation) to the repeated registration (**Figure B.2**), only moderately increased deviations in data shift is seen. Most notably, systematic dilation of the aortic mask generated the largest shift, albeit the effects were still small with variability of -2.7 ± 9.2 mmHg/m, and still with strong agreement (r = 0.95, p < 0.001).

For MSDR, repeat co-registration alone generated a mean shift of -180.7 ± 413.2 cm/sm^3^ (see **Figure B.1**), with a linear regression coefficient of r = 0.91, p < 0.001. When adding the systematic segmentation variations to the repeated registration (**Figure B.3**), increased MSDR variations were observed, with the greatest degree of variability seen with eroded segmentation masks (mean difference of -132.8 ± 685.2 cm/s^3^).

The reported variability of FL EF, FL ΔP_max_ and MSDR should be contrasted to the degree of average separation of these metrics between enlarging and stable groups reported in the main results (e.g., 39% for FL EF, 24.9 mmHg/m for FL ΔP_max_, and 382.9 cm/s^3^ for MSDR ). The degree of interobserver variability is far lower than the average separation in groups for FL EF and FL ΔP_max_.


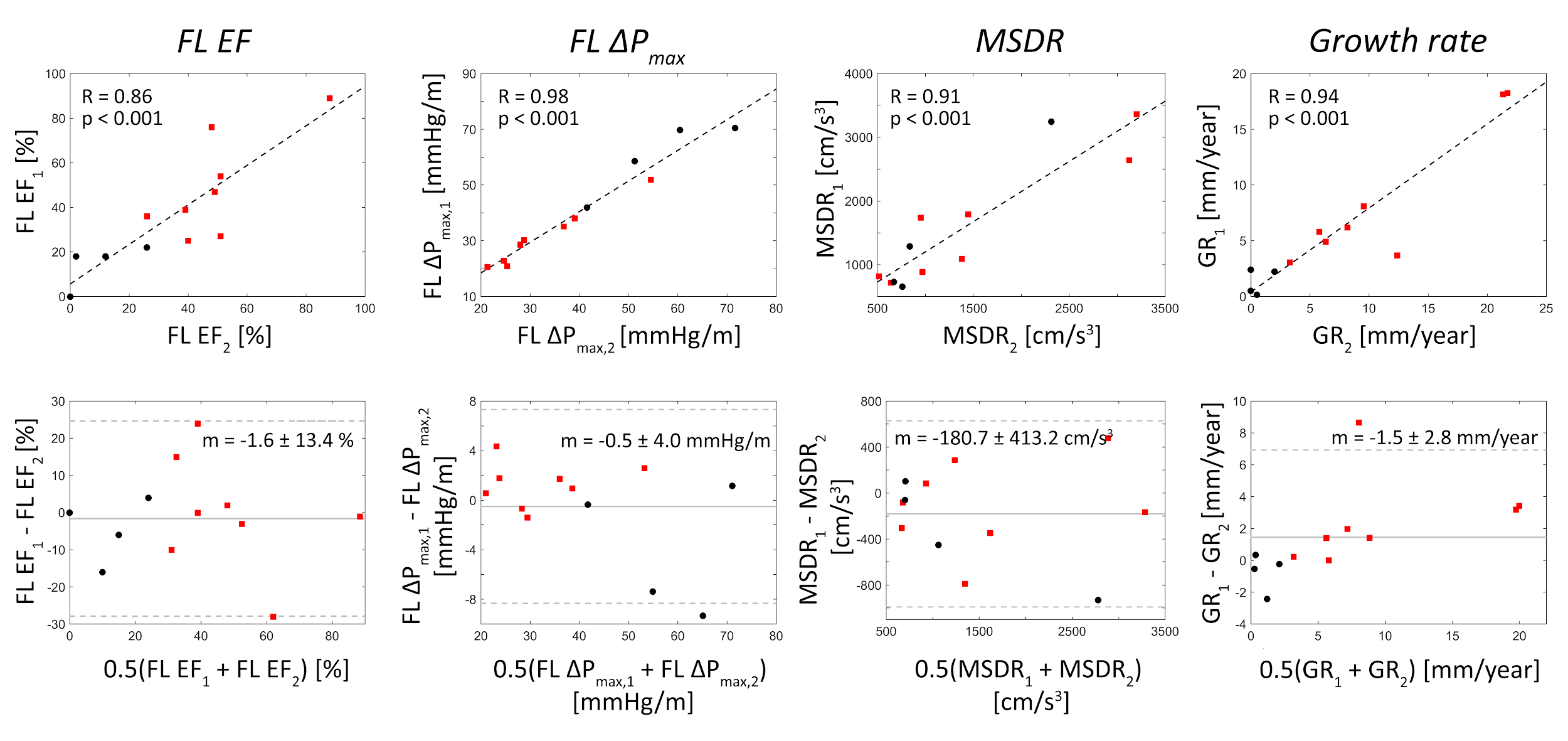


**Figure B.1**: Results from the reproducibility analysis, shown as linear regression (top row) and Bland-Altman plots (bottom row) for multiple readings of, from left to right columns: false lumen ejection fraction (FL EF), false lumen maximum relative pressure (FLΔP_max_) with repeated mask registration, maximum systolic deceleration rate (MSDR) with mask registration, and aortic growth rate (GR, assessed by maximum aortic diameter), respectively. In each plot, data is shown for both subjects with stable (black) and enlarging (red) aortic dimensions.


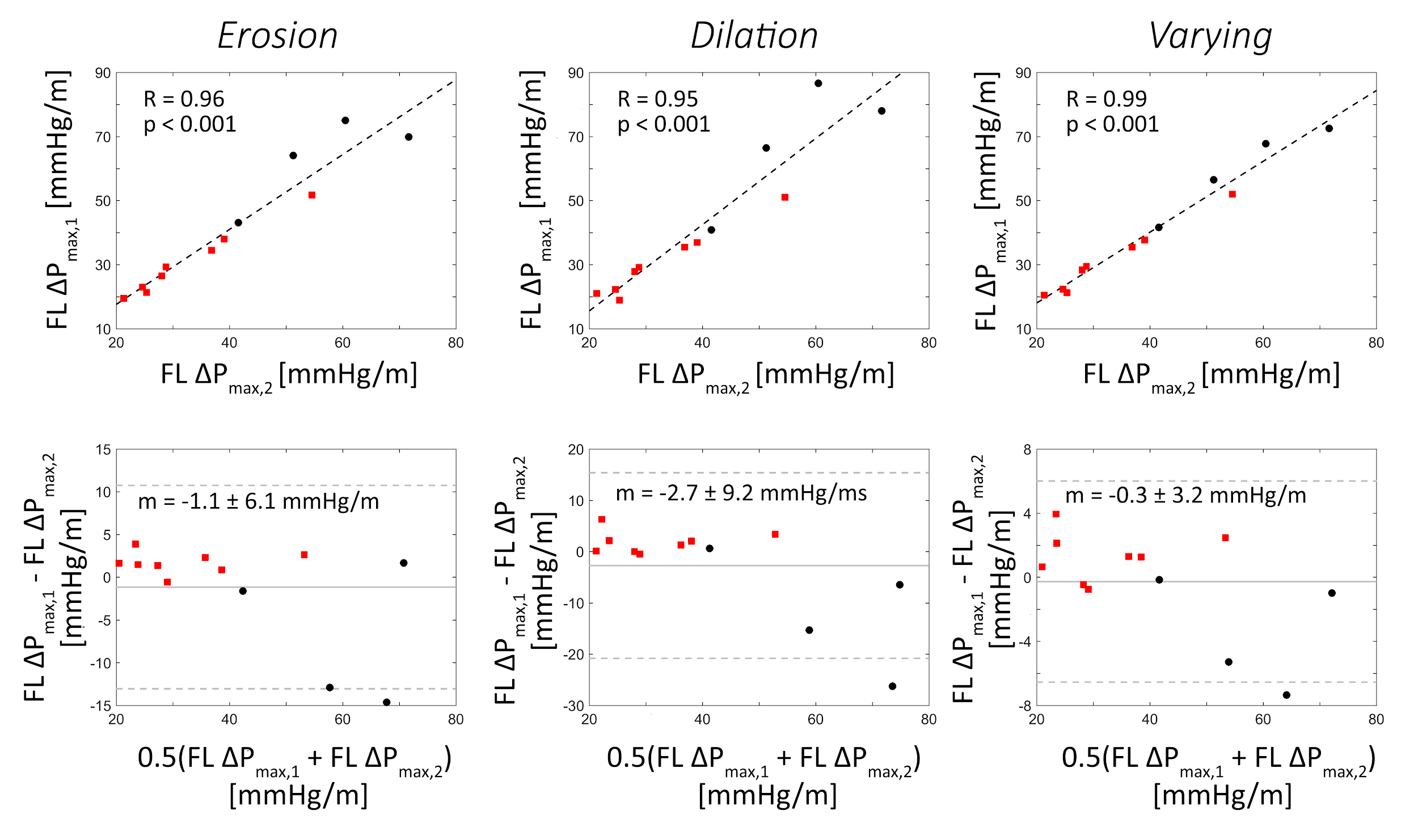


**Figure B.2**: Isolated reproducibility analysis for the derivation of false lumen maximum relative pressure (FLΔP_max_). Here, in addition to the new mask registrations (with results shown in Figure A.1), typical classes of segmentation variations are incorporated including systematically smaller segmentations (Erosion), systematically larger segmentations (Dilation), and segmentations varying along the length of the aorta (Varying). In each plot, data is shown for both subjects with stable (black) and enlarging (red) aortic dimensions.


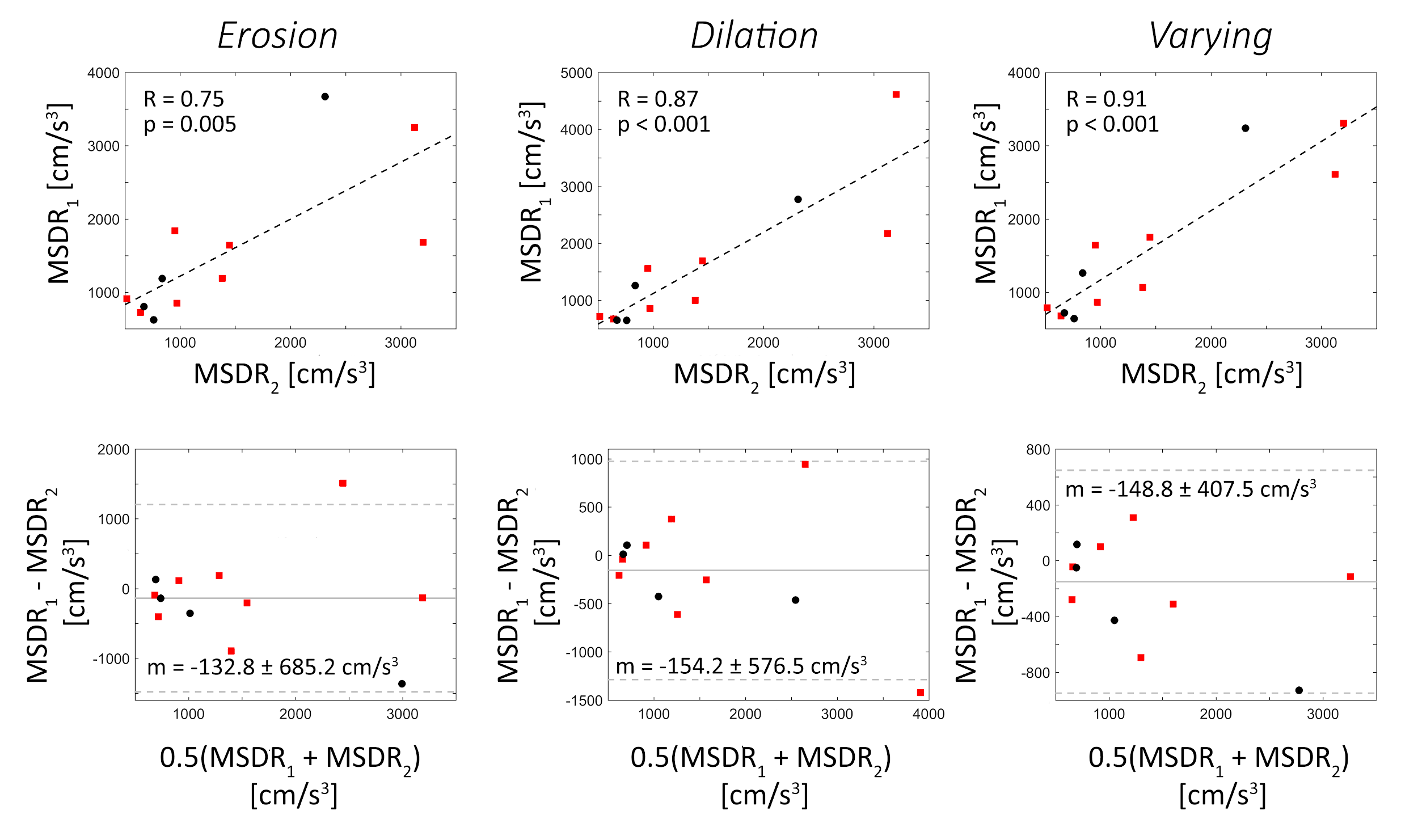


**Figure B.3**: Isolated reproducibility analysis for the derivation of maximum systolic deceleration rate (MSDR). Here, in addition to the new mask registration (with results shown in Figure A.1), typical classes of segmentation variations are incorporated including systematically smaller segmentations (Erosion), systematically larger segmentations (Dilation), and segmentations varying along the length of the aorta (Varying). In each plot, data is shown for both subjects with stable (black) and enlarging (red) aortic dimensions.

**Table A.1**: Reproducibility analysis - evaluating interobserver variability. Note that for false lumen maximum relative pressure (FLΔP_max_) and maximum systolic deceleration rate (MSDR) - both relying on false lumen co-registration and segmentation - data are presented for new co-registration only, as well as additional variation of segmentation (varying, smaller, larger, respectively).

| **Characteristics** | **Pearson’s correlation statistics (*R; p*)** | **Limits of agreement  (*m ± sd*)** |
| --- | --- | --- |
| *FL EF (%)* | 0.86; <0.001 | -1.6 ± 13.4 |
| *FL maximum relative pressure (mmHg/m)* |  |  |
| *Co-registration* | 0.98; <0.001 | -0.5 ± 4.0 |
| *Varying* | 0.99; <0.001 | -0.3 ± 3.2 |
| *Smaller* | 0.96; <0.001 | -1.1 ± 6.1 |
| *Larger* | 0.95; <0.001 | -2.7 ± 9.2 |
| *MSDR (cm/s^3^)* |  |  |
| *Co-registration* | 0.91; <0.001 | -180.7 ± 413.2 |
| *Varying* | 0.91; <0.001 | -148.9 ± 407.5 |
| *Smaller* | 0.75; 0.005 | -132.8 ± 685.2 |
| *Larger* | 0.87; <0.001 | -154.2 ± 576.5 |
| *Aortic growth rate (mm/year)* | 0.94; <0.001 | 1.5 ± 2.8 |
